# An Account of the Most Frequented Watering Places on the Continent, &c.

**Published:** 1837-01-01

**Authors:** 


### AN ACCOUNT OF THE MOST FREQUENTED WATERING-PLACES ON THE CONTINENT, AND OF THE MEDICINAL APPLICATION OF THEIR MINERAL SPRINGS; with Tables of Analysis, and an Appendix of English Mineral Waters. By *Edwin Lee*, Esq. M.R.C.S. Author of "Observations on Continental Medical Institutions and Practice," "Treatise on Nervous Disorders," &c. 12mo, pp. 232. London, Longman and Co. 1836.

SINCE the Continent has been opened to the English, John Bull, Mrs. Bull, and all the little Bulls, have taken a wonderful liking to the various mineral waters so plentiful in France and Germany. That many of these waters, as well as those of our own country, have acquired celebrity from their own intrinsic merits, we do not doubt; but that their efficacy has been chiefly indebted to various well-known auxiliaries, is now universally acknowledged. Be this as it may, the watering-places of England and Continental Europe will be annually visited by shoals of our countrymen, and as there is scarcely a medical practitioner, of any standing, who is not, every year, consulted by patients respecting foreign medicinal waters, as well as domestic, it behoves him to acquire a general knowledge of their qualities, if he wishes to avoid the suspicion of ignorance.

The work before us is certainly the most compendious one of the kind which we now possess, especially as relates to Continental watering-places, with this advantage, that it is the most recent, and that the author has himself visited the places described, and observed the effects of the waters on the human constitution. If we were inclined to find fault, it would be with the brevity of the reports. The profession could surely afford to buy and consult a much larger volume than this on so important and extensive a subject. The Appendix, on English mineral waters, is still more open to the above objection of brevity—

——— dum brevis esse laboro
Obscurus fio.

Mr. Lee has classed mineral waters under six heads—sulphureous—chalybeate—saline thermal—saline aperient—alkaline—acidulous springs; to which he has added an Appendix on English mineral springs. The work is prefaced by a discourse on mineral waters in general, and on the greater attention which is paid to this part of hygiene and therapœia on the Continent than in England.

Under each class, the principal waters are indicated, with a short description of the localities in which they are situated—the accommodations for patients—and the diseases to which the waters are applicable. We shall give a sketch of the most celebrated and most frequented.

1. *Sulphureous Springs*. Amongst the most powerful waters of this class, are AIX-LA-CHAPELLE—Baden (near Vienna)—Harrowgate—and the Pyrennees. Used in the form of bath, their primary operation is on the skin—their secondary, on the mucous membranes and viscera. Taken internally, they act on the bowels, the air-passages, and the urinary organs. In cutaneous and rheumatic affections, they are often very efficacious. The springs of Aix are of very high antiquity. The place itself is very pleasant. One of the springs is at a temperature of 130° Fahr. containing a large quantity of sulphur. There are also some chalybeate waters here.

"A course of these waters is serviceable in most cases where the object is to act gradually, though powerfully, upon the skin, mucous membranes, abdominal viscera, nervous and absorbent systems; as in obstinate rheumatic or gouty affections, with calcareous concretions in the joints; contraction or loss of power of the limbs; paralytic cases, when not dependent upon cerebral disease; intestinal and hepatic inactivity; piles; hypochondriasis; disordered health from residence in unhealthy climes, abuse of mercury, or other causes; some cases of neuralgia, when not attended with great irritability of the system; asthma and chronic bronchitis, with increased secretion, especially in old persons; some chronic diseases of the skin, as scabies, impetigo, psoriasis, lepra, and prurigo; enlargement of the lymphatic glands; vesical catarrh, with tendency to the formation of stone; leucorrhœa, and other mucous discharges of long duration. Dr. Alertz is one of the most esteemed among the resident physicians." 40.

These waters contain sulphur, muriate of soda, sulphate of soda, carbonate of soda, phosphate of soda, and a few other ingredients—in atomic doses—31 grains of solid matter in the pint.

### Baden

Is a clean little town near Vienna, at the foot of the Styrian Mountains. Gaming is wisely prohibited by Government. The springs are hot—some of them considerably so—and are rather strongly saline and sulphureous. They resemble those of Aix-la-Chapelle, but are rather weaker. The waters are more used for bathing than for drinking.

### Aix—(in Savoy).

These waters, near Chambery, are of high antiquity, and situated in a beautiful country. The sulphur spring, and the so-called alum spring, are much frequented. The diseases for which they are prescribed, are those to which sulphureous springs are applicable.

### Sulphureous Springs of the Pyrennees.

The Valley of the French Pyrennees presents a greater abundance, in the same space, of mineral springs, than any other place in the world. They also present more *agrémens* for a Summer residence than most other localities—"more resources for bodily and mental recreation." The accommodations are excellent, and living is not expensive. The scenery is various and beautiful.

### Bareges.

This village, consisting of only 80 houses, is situated on the verge of a mountain torrent, in a cheerless spot, enclosed by steep, pine-covered, and snow-tipped mountains. It is crowded during the season with persons of all ranks, and offers better accommodations than might be expected. There are six springs, slightly sulphureous in taste.

" The water is perfectly clear, and does not taste strongly of sulphur; but the smell is very decided. It is mineralised principally by the sulphuret of sodium; but also contains carbonate of soda, a small quantity of sulphate and muriate of soda, azote, sulphuretted hydrogen, and glairine or animal matter. Taken internally, it often produces, like other sulphurous waters, a degree of excitation, marked by acceleration of the pulse, perspiration more or less abundant, increased appetite, and sometimes sleeplessness. It is not in general purgative, and even sometimes induces constipation, particularly when exclusively used for bathing; but is diuretic, diaphoretic, and expectorant. By its local or general stimulating properties, it cleanses foul ulcers, lessens the induration of callous and fistulous sores, promotes the exfoliation of carious portions of bone, and cicatrization, frequently causing foreign bodies which had long been imbedded in the deeper textures to advance towards the surface. It is also highly efficacious in allaying long-existing pains, whether of a rheumatic nature or arising from wounds; in remedying the stiffness and immobility of joints when these symptoms depend upon tumefaction of the soft parts; in hemorrhoidal affections, jaundice, and chronic disorders of the chylopoietic viscera, especially long-standing dysentery; in chronic syphilitic diseases, and those resulting from the abuse of mercury; in strumous swellings, dry asthma, and chronic bronchitis, when not attended by much secretion. In cases of humid asthma, the water at Cauterets generally agrees better than that of Baréges." 54.

### BAGNIERES DE LUCTION

Is a watering-place of very agreeable locality, and much frequented. Some of the waters are 58° or 59° of Reaumur, which is rather high. They contain hydro-sulphuret of sodium—carbonate of soda—some muriate and sulphate of soda, and silex. The waters are used internally and externally. They often occasion constipation of bowels, and require aperients. In rheumatic and cutaneous affections—in psoriasis and eczema—these waters are said to be more powerful than those of *Baréges*.

2. *Chalybeates*. The most celebrated of these is PYRMONT, eight German miles from Hanover, situated in a delightful valley, and containing 2000 inhabitants. The accommodations are good, and the amusements numerous. Pyrmont possesses three different kinds of springs—chalybeate—saline—and acidulous. The chalybeate, however, is that which has immortalized Pyrmont. From this spring, upwards of one hundred thousand flasks are annually exported.

" The water is exceedingly rich in iron and carbonic acid gas, is limpid, very sparkling, of an agreeably acid and somewhat astringent taste, and on standing, deposits a brownish sediment, composed of oxide of iron and manganese; its temperature is 10° R. A few glasses taken in quick succession occasion a sort of temporary intoxication, with a feeling of satisfaction and hilarity. When a certain quantity is drunk it has an aperient effect, and promotes the excretion of urine. It is better supported than most waters of this class; and when it can be procured, merits a decided preference over others, especially in cases of general debility remaining after loss of blood, copious discharges, parturition, or severe illness; in chlorosis, hypochondriasis, hysterical and other nervous affections; diseases of the digestive and urinary apparatus, depending on general or local debility; passive uterine and hemorrhoidal hemorrhage; suppressed or difficult menstruation; leucorrhœa, and sterility from weakness, especially where the generative organs had not acquired their full development previous to marriage." 84.

SPA,

Which is second only to Pyrmont, is now comparatively deserted. It is out of favour with the English, and also with the Dutch.

LANGEN-SWALBACH

Has been so celebrated by Captain Head, in his Brunnens, that we need scarcely notice it here.

KISSENGEN.

This watering-place, situated in Bavaria, is coming into great note. It has been frequented, indeed, for 300 years past. Upwards of a thousand annual visitors have been there of late years. Two of the springs are chalybeate—the third acidulous. The water is exported in large quantities. It is of a yellowish colour, and ferruginous saline taste. The astringent quality of the chalybeate is, in a great measure, neutralized by the salts which it contains.

"The action of these springs upon the mucous membranes, the absorbent and nervous systems, renders them peculiarly applicable to cases where it is desirable to produce a tonic, but not astringent effect, as in diseases of the chylopoietic viscera dependent upon want of tone; hypochondriasis and hemorrhoids, with tendency to constipation; chronic affections of the mucous membrane of the air-passages, or of the urinary organs, when without inflammatory complication; leucorrhœa, and irregular menstruation arising from general or local debility; scrofulous cases; many nervous complaints, and other cases to which the exhibition of tonics is suited. The baths are said to be very efficacious in cases of atonic gout and rheumatism." 95.

MARIENBAD

Is another of the lions of Germany, lying about five German miles from Carlsbad, in a valley surrounded by high mountains. It possesses two kinds of mineral springs—one saline alkaline—the other alkaline chalybeate. The latter is much used, where chalybeates are proper. It is rich in carbonic acid, and the iron is intimately combined with it. It is, therefore, easily borne. It is exciting and strengthening.

SALINE THERMAL SPRINGS.

Wisbaden is amongst the foremost of these. It is but an hour's drive from Mayence, lying in a valley encircled by low hills. Sunday is the grand gala day, though the population is Protestant, and then the shops, the theatres, ball-rooms, &c. are thronged with visitors from Mayence, Frankfort, &c. The springs of Wisbaden were used by the Romans, and lately they have been much frequented by the English.

"The Kochbrunnen, or boiling spring, is the most generally used; it rises in the town, and is the central point where a crowd of persons of various nations assemble at stated hours to sip their glasses of water, while sauntering about under the acacia avenues, and listening to the musical band. The water is perfectly limpid when taken into a glass; its taste is rather agreeable than otherwise, and has been compared to that of weak broth oversalted; its temperature is 151° Fahrenheit. The carbonic acid gas is seen bubbling up to the surface of the water; the quantity contained in a pint amounts, according to Ritter, to 6¾ cubic inches. This spring holds in solution a greater quantity of saline substance than any other of the same class; those of Pyrmont and Borcette, perhaps, excepted."

"Used in the form of bath, the water is generally exciting; it stimulates powerfully the skin and absorbent system, not unfrequently producing an eruption on the surface, whence the excitement is transmitted to internal parts, especially the abdominal viscera, increasing the activity of their functions, though in many cases no perceptible change is experienced at the time. Internally taken it promotes digestion, sometimes producing an aperient effect; frequently increases the secretion of the kidneys, and acts consecutively upon the skin. Most invalids combine the internal with the external use of the water.

A course of this water is specially applicable to cases of articular rheumatism, with swellings of the joints of long duration; chronic gout, particularly when accompanied with calcareous deposits; disorder of the digestive powers, with vitiated secretion; strumous enlargement of the glands, or disease of bones; derangement of the general health in persons who have long resided in tropical climates, as well as that caused by intemperance, or the abuse of mercury, when not attended by exceeding debility; some cases of neuralgia and tic; amenorrhœa and dysmenorrhœa, if unaccompanied with a high degree of local irritation; paralytic affections, especially if caused by morbid impressions upon the organic nervous system, and not depending upon cerebral disease; and some chronic diseases of the skin. It is also said to be efficacious in bronchial complaints and asthma with copious expectoration; in these cases the inhalation of the vapour is joined to the employment of the water. Douches, local and general vapour-baths, are used in many cases of local disease, as is also occasionally the muddy sediment deposited from the water.

*Solid Substance yielded by a Pint of Water from the Kochbrunnen, analysed by Kastner.*

| | Grains. |
|---|---|
| Muriate of Soda | 44·225 |
| Sulphate of Soda | 0·700 |
| Muriate of Lime | 5·480 |
| Sulphate of Lime | 0·420 |
| Carbonate of Lime | 1·650 |
| Muriate of Magnesia | 0·790 |
| Carbonate of Magnesia | 0·700 |
| Extractive Matter | 1·750 |
| Iron | 0·078 |
| Muriate of Potass | 1·200 |
| Fluate of Magnesia | 1·600 |
| | 57·593." |

### Baden-Baden.

These waters are very nearly the same as those of Wisbaden. The situation is very beautiful, and excellent hotels and table-d'hôtes render this fashionable watering-place one of great resort by all nations. We were sorry to see gaming carried on there to a shameful and ruinous extent.

### Vichy.

We must now conclude our notice of Mr. Lee's book, with some account of this now celebrated mineral spring. It is situated in an extensive valley, 87 leagues from Paris, with excellent accommodations. The springs were used by the Romans, and many antiquities are still discovered there. The temperature of the different springs varies from 18 to 34 of Reaumur.

"The water of Vichy is limpid, without smell, of an acidulous alkaline taste, not unlike that of soda water. It contains a large proportion of free carbonic acid, which is most abundant in the Fountain des Acacias (twenty-three cubic inches to the pint). Next to this spring, Lucas, Les Célestins, and Le Grand Bassin, are the richest in this respect. Carbonate of soda, and carbonic acid gas are the predominating ingredients of Vichy water: this salt is extremely plentiful in the Célestins, which contains ninety-six grains to the pint; the Lucas likewise contains a large quantity. Iron is also found in these springs; the Acacias contains half a grain to the pint, and might be ranked among the chalybeate springs, were it less rich in saline substance.

The operation of these waters is alterative, laxative, and diuretic, without being aperient or diaphoretic: it affects most perceptibly the secretion of urine, increasing its quantity, and altering its quality, so as, according to M. D'Arcel, to render alkaline the urine of a person after having drank three or four glasses, or taken a bath: hence it is highly useful in some diseases of the urinary organs especially stone, and the disposition to the formation of lithic acid, or what is commonly called red gravel. The water is also much used, both at the springs and throughout France, in those deranged states of the digestive functions which are termed abdominal engorgement or obstruction; in chronic enlargement of the liver or spleen; long standing stomach disorder, with acidity; hemorrhoidal affections; and uterine derangement. It has but little effect on scrofula, most diseases of the skin, and rheumatism, and often aggravates gout. Its use should be prohibited to plethoric, or highly irritable persons, to those of rigid fibre, in nervous diseases and affections of the chest." 185.

We have now given sufficient specimens of Mr. Lee's book to enable our readers to judge for themselves. It is certainly a useful compendium, both for the home practitioner and the wandering invalid.

